# 2093. Utilizing Implementation Science to Identify Barriers and Facilitators to Implementing Harm Reduction Services in the Veterans Health Administration (VHA)

**DOI:** 10.1093/ofid/ofac492.1715

**Published:** 2022-12-15

**Authors:** Leah Harvey, Samantha K Sliwinski, Kimberlee Flike, Jacqueline Boudreau, Allen L Gifford, Westyn Branch-Elliman, Justeen Hyde

**Affiliations:** Boston Medical Center and Boston University School of Medicine, Boston, Massachusetts; Veterans Affairs Boston Center for Healthcare Organization and Implementation Research, Boston, Massachusetts; Veterans Affairs Boston Center for Healthcare Organization and Implementation Research, Boston, Massachusetts; Veterans Affairs Boston Center for Healthcare Organization and Implementation Research, Boston, Massachusetts; Veterans Affairs Boston Center for Healthcare Organization and Implementation Research, Boston, Massachusetts; VA Boston Healthcare System, Boston, MA; Veterans Affairs Boston Center for Healthcare Organization and Implementation Research, Boston, Massachusetts

## Abstract

**Background:**

Rising rates of substance use, particularly synthetic opioids, have led to increases in fatal overdoses and injection-associated infections. Harm reduction, including infection prevention via provision of supplies and education, is an approach to minimize risk of severe outcomes. Although harm reduction services (HRS) are highly evidence-based, implementation in most healthcare settings is limited. The aim of this study was to identify facilitators and barriers to the implementation of HRS to inform strategies for increasing access and adoption of a comprehensive bundle of harm reduction resources within the VHA.

**Methods:**

Qualitative interviews were conducted using a semi-structured interview guide and explored how harm reduction is currently understood and implemented by VHA providers and was designed to identify perceived gaps and barriers. Data were analyzed using a directed content analysis. After barriers and facilitators were identified, they were mapped to relevant implementation strategies using the Consolidated Framework for Implementation Research - Expert Recommendations for Implementing Change (CFIR – ERIC) tool.

**Results:**

15 interviews with VHA providers (physicians, social workers, pharmacists, and directors of addiction and mental health services) were conducted across 5 sites. Multiple barriers and few facilitators to the provision of HRS were identified (Table 1). Currently, HRS were thought to be fragmented and dependent on the knowledge, time, and comfort level of individual providers. Participants also highlighted stigma around substance use, limited support, and burdensome regulatory requirements. Existing infrastructure, social programming, and local champions were highlighted as facilitators. Given these factors, implementation strategies that may be bundled to promote adoption of HRS include engagement of champions, communications and educational strategies, existing policies, and creation of dashboards and tracking and feedback systems (Table 1).

**Figure:**
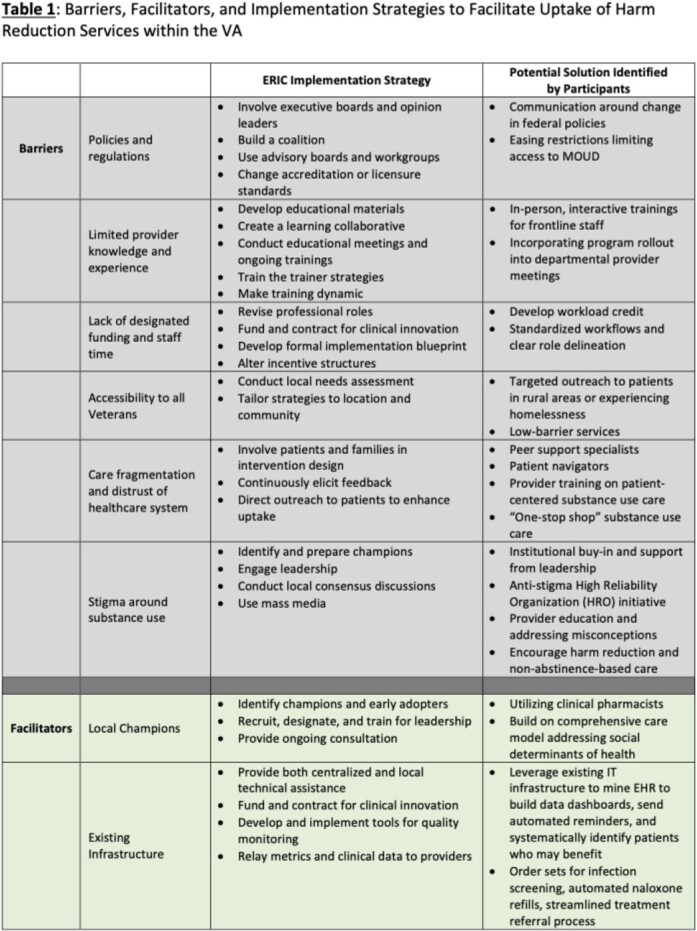

**Conclusion:**

HRS are effective, evidence-based, and patient-centered tools. Mapping of barriers to evidence-based implementation strategies may help improve integration of HRS into VHA healthcare, however, challenges addressing stigma remain a substantial barrier.

**Disclosures:**

**Westyn Branch-Elliman, MD, MMSc**, DLA Piper,LLC/Medtronic: Advisor/Consultant|Gilead Pharmaceuticals: Grant/Research Support.

